# Black Nickel Coating as a Broadband Terahertz to Deep-Ultraviolet Absorber

**DOI:** 10.1021/acsomega.5c12663

**Published:** 2026-02-03

**Authors:** Aadya Menon, Hanna Maltanava, Nikita Belko, Maria Cojocari, Mikhail Gorbun, Aleksandr Saushin, Konstantin Tamarov, Jari T. T. Leskinen, Mikko Selenius, Sari Suvanto, Dmitry Semenov, Sergei Malykhin, Vesa-Pekka Lehto, Georgy Fedorov, Polina Kuzhir

**Affiliations:** † School of Computing, 163043 University of Eastern Finland, Joensuu 80101, Finland; ‡ Department of Physics and Mathematics, University of Eastern Finland, Joensuu 80101, Finland; § Department of Technical Physics, University of Eastern Finland, Kuopio 70211, Finland; ∥ Department of Chemistry and Sustainable Technology, University of Eastern Finland, Joensuu 80101, Finland

## Abstract

Broadband absorbers capable of attenuating electromagnetic radiation from terahertz (THz) to deep-ultraviolet (DUV) frequencies are critical components in spectroscopy, imaging, sensor technology, and energy harvesting systems. However, most conventional absorbers are limited by their narrow operational bandwidth and often require complex or costly nanostructuring. In this study, we present a black nickel (b-Ni) coating fabricated via scalable electrodeposition onto copper substrates, followed by controlled acid etching, as a cost-effective and robust broadband absorber. The resulting b-Ni films, characterized by scanning electron microscopy (SEM) and X-ray photoelectron spectroscopy (XPS), demonstrate ultrabroadband absorption spanning from 30 to 1500 THz, with absorptivity exceeding 95% throughout this range. Compared to nanostructured metamaterials and moth-eye analogs, the b-Ni coating offers significant advantages in fabrication simplicity and scalability. This work positions chemically etched b-Ni coatings as a highly promising material for large-area applications in spectral and imaging instrumentation, including emerging systems operating within the THz frequency range.

## Graphical abstract

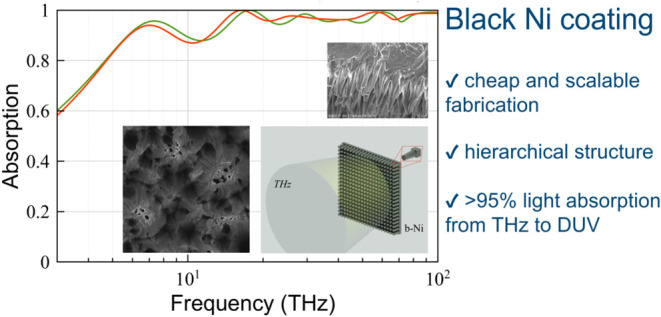

## Introduction

In quantum technologies and THz photonics, broadband absorbers are crucial for thermal management and radiation shielding. Coatings that reduce reflections and boost absorption enhance bolometers, photodetectors, and sensors,^1,2^ and are used in aerospace and defense to reduce radar and infrared signatures.^3^ Their wide spectral absorption also benefits energy harvesting systems like thermophotovoltaics and solar thermal collectors, where higher absorption improves performance.^4,5^ In instruments operating from THz to UV, scattered light increases detector noise and reduces sensitivity, which black light-absorbing coatings help mitigate.^6^

Several classes of materials are commonly used to prepare strongly absorbing coatings. Metal-black coatings, typically composed of gold,^7^ silver,^8,9^ or tungsten,^10^ have been extensively studied for strong absorption, but it is typically measured in a relatively narrow spectral range, while high cost of such materials limits their practical use. Similarly, vertically aligned carbon nanotube arrays exhibit extremely low reflectivity (0.045%), but suffer from adhesion and processing challenges.^11,12^ Metamaterial-based absorbers can also achieve over 90% absorbance from near-UV to near-IR; however, their intricate nanofabrication processes hinder scalability and cost-effectiveness.^13,14^ As alternatives, black silicon structures fabricated through wet chemical etching, maskless reactive ion etching, or picosecond laser irradiation can achieve reflectivity as low as 3–4%, though these methods involve complex and time-consuming processing steps.^15–17^ Recently, silicon moth-eye structures coated with thin graphitic films have demonstrated ultrabroadband absorption exceeding 98% from THz to DUV.^18,19^ Finally, recent studies have explored topological and reconfigurable metadevices^20^ and materials based on 1T-phase semimetals such as TaS_2_ ^21^ and CoTe_2_ ^22^ for precise control of THz radiation.

Nickel and its alloys are also among the materials frequently used as substrates for the preparation of black coatings.^23–27^ These coatings are widely used in various fields,^28–30^ including space instruments such as spacecraft orientation sensors and efficient selective coatings.^31,32^ Key requirements for light-absorbing coatings include high strength, corrosion resistance, controlled reflectance and brightness coefficient, and very high absorptivity.^33^ Nickel and its alloys, especially black Ni–P coatings, meet these needs with high absorptivity, low reflectance, and strong mechanical and chemical stability in the optical and near-IR ranges.^34^ By tuning electrodeposition and postprocessing conditions (e.g., acid etching), Ni–P alloys yield black coatings with over 90% solar absorbance and high environmental stability, making them efficient solar absorbers.^23,26,28,35^ Despite these promising properties, the light absorption of black Ni coatings has not yet been investigated across a broad spectral range.

Previous studies indicate that black coatings can exhibit excellent optical properties. However, they often suffer from high cost and complex fabrication processes, or their strong light absorption is limited to a narrow spectral range. In this work, we propose a method to fabricate a black coating that combines a simple, scalable fabrication process with strong light absorption across an ultrawide spectral range, spanning from THz to DUV (30 to 1500 THz). The near perfect absorber is produced via electrodeposition of Ni, followed by acid etching. The morphology and chemical composition of the material are characterized using SEM, energy-dispersive X-ray spectroscopy (EDS) mapping, and XPS. We also compare the thermal emission spectra of the b-Ni coating and the previously reported Si moth-eye structure in the 3–35 THz range.^18,19^ In addition to our experimental approach, we conduct full-wave simulations using COMSOL Multiphysics, which reveal that the broadband absorption originates from the hierarchical structure of the b-Ni coating, combining micron-scale cone-like features with a nanostructured surface.

## Experimental Section

### Preparation of the b-Ni Coating

Copper foil samples (C12500 alloy) with dimensions of 25 × 25 mm^2^ and a thickness of 40 μm were used as substrates for the electrodeposition of b-Ni coatings. Before plating, the substrates were degreased with acetone, rinsed with deionized water, and etched in an aqueous solution containing 1300 g/L H_3_PO_4_ and 350 g/L NH_4_NO_3_, at room temperature for 30–40 s. After etching, the substrates were rinsed with deionized water and prepared for the b-Ni electrodeposition process. Due to the low pH of the deposition electrolyte, no additional surface activation of the copper was necessary. The electrolyte used for b-Ni deposition contained 180 g/L NiSO_4_·7H_2_O, 10 g/L NiCl_2_·6H_2_O, 10 g/L H_3_PO_4_, 14 g/L KH_2_PO_4_, 20 g/L H_3_PO_3_, and 2 g/L saccharin. The Ni salts supplied nickel for electrodeposition, while the phosphate and dihydrophosphate provided buffering properties. Phosphite acted as a source of phosphorus for the coating, and saccharing was added to control the electrodeposition process and enhance the properties of the resulting coating. The pH of the electrolyte was 2. All chemicals were of analytical or chemical grade. Electrodeposition was carried out in a thermostatically controlled electrochemical cell at 60 ± 2 °C, with cathode agitation at a frequency of 0.5 Hz. The cathodic current density was maintained at 30 mA/cm^2^, and the process lasted approximately 2 h, resulting in a coating thickness of 45 ± 5 μm. The pH of the electrolyte was maintained at 2 during deposition by adding concentrated sulfuric acid. Finally, the coatings were etched by immersing the coated samples in a 5 M nitric acid solution at 55 ± 1 °C for 160 s.

### Characterization Techniques

The morphology of the prepared b-Ni coating was studied using a Hitachi S-4800 field emission scanning electron microscope with an EDS accessory and a Zeiss LEO 1550 scanning electron microscope.

XPS spectra were acquired using a Thermo Scientific Nexsa G2 spectrometer with Al K_α_ radiation and a 400 μm X-ray spot size. Charge compensation was achieved using an electron flood gun operating at an emission current of 100 μA and an extractor voltage of 40 V. Low-resolution survey spectra were recorded with a pass energy of 200 eV, a step size of 1 eV, and a dwell time of 10 ms. Selected peaks were then analyzed in high-resolution mode using a pass energy of 50 eV, a step size of 0.1 eV, and a dwell time of 50 ms. High-resolution spectra were used for quantitative elemental analysis and chemical state determination. Peak fitting was performed using Thermo Scientific Avantage software (version 6.9).

### Optical Measurements

The absorptivity of the b-Ni coating in the 3 to 35 THz range was evaluated by measuring the emission spectra of the coating and a reference sample using a custom-made FTIR setup. As the reference sample, we used a carbon-coated moth-eye Si structure, which acts as a near-perfect blackbody across the far-IR and THz ranges.^18,19^ By dividing the emission spectrum of the b-Ni coating by that of the reference sample, we obtained the spectral absorptivity of b-Ni. Both samples were heated at 60 °C. According to Kirchhoff’s law, the thermal emission of a body, *I*_*TH*_(*T*, ω), is a product of the blackbody emission spectrum, *I*_*BB*_(*T*, ω), given by Planck’s law and the absorptivity of the body, *A*(ω):^36^

1 $$I_{\mathrm{TH}}\left(T,\omega \right)=I_{\mathrm{BB}}\left(T,\omega \right)A\left(\omega \right)=\frac{\hbar \omega^{3}}{4\pi^{2}c^{2}}\frac{1}{\exp\left(\frac{\hbar \omega }{k_{B}T}\right)-1}A\left(\omega \right)$$

During the emission measurements, radiation was collected from directions deviating from the sample normal by up to 12 °.

Reflectivity spectrum for the b-Ni coating was measured in two spectral ranges using different techniques: 12–120 THz using FTIR, and 130–1500 THz using diffuse reflectance spectroscopy (DRS). The FTIR spectrum was acquired using a Bruker α FTIR spectrometer with a diffuse reflectance accessory (A241/D) and a golden mirror used as a reference sample. The DRS spectrum was measured using a PerkinElmer Lambda 1050 spectrophotometer equipped with a 150 mm InGaAs integrating sphere. Reflectivity was then recalculated into absorptivity using the expression

2 α=1−r

under the assumption that the transmittance of a 40 μm-thick layer of copper is zero.

## Results and Discussion

### Structure and Composition of the Black Ni Coating

First, the structure of the prepared b-Ni coating was studied using SEM. As shown in the top-view SEM micrographs (Figure 1A,B), the fabricated coating exhibits a nodular morphology characterized by irregularly shaped, cone-like structures with pores between them. The cross-sectional image (Figure 1C) reveals a multilayered architecture comprising a copper support layer, an intermediate metallic nickel layer, and cone-like features etched into the nickel. These cones have base diameters of approximately 5 μm and heights reaching ∼10 μm. A higher-magnification micrograph (Figure 1D) reveals that the cones are porous, with a rough surface texture composed of nanoscale features around 50 nm in size.

**Figure 1:**
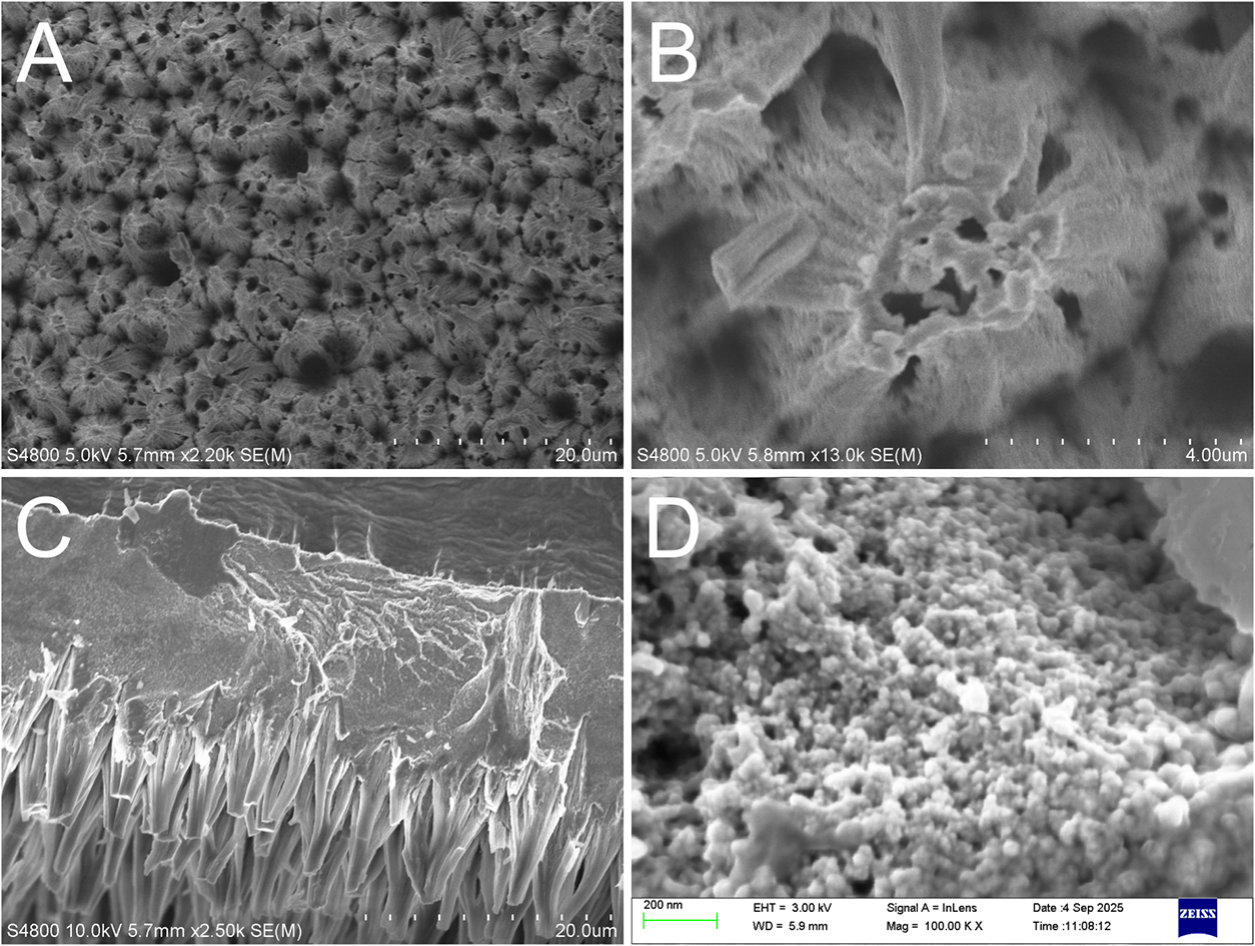
SEM microphotographs of the b-Ni coating. (A, B) Top-view images at lower magnification (the acceleration voltage was 5 kV); (C) Cross-section image (the acceleration voltage was increased to 10 kV to enhance the contrast between the copper and nickel layers); (D) Top-view image at higher magnification (an in-lens secondary electron detector was used in conjunction with an acceleration voltage of 3 kV to achieve higher resolution and enhance the topography of the surface).

Elemental distribution within the b-Ni coating was analyzed via EDS mapping. The results indicate that the bulk of the cone-like structures consists of metallic nickel, which is enveloped by a thin oxidized surface layer with a thickness of less than 1 μm (Figure S1). The thickness of the oxidized layer progressively increases from the base toward the tip of the cones.

The elemental composition and chemical state of the coating were further studied using XPS. The elemental abundances are summarized in Table 1. The measurements revealed a Ni content of only 6.5%. This value represents the composition of a thin surface-near layer, as determined by XPS analysis, and aligns with the EDS mapping results, which indicate that the metallic nickel within the cone-like structures is coated by an oxidized layer. In addition to Ni, the coating contained O, C, N, and P, with oxygen and carbon being the most abundant elements, each contributing approximately 40%.

**Table 1:** Elemental Composition of the b-Ni Coating Determined by XPS

peak	atomic fraction (at %)
O 1s	41.8
C 1s	41.7
N 1s	3.7
P 2p	6.4
Ni 2p	6.5

High-resolution XPS spectra are presented in Figure 2. The O 1s XPS spectrum was fitted with a single peak at 532.8 eV, assigned to C–O–C/O–C = O and/or P–O–P environments.^37,38^ The C 1s spectrum contained peaks at 285.7 and 288.5 eV, respectively ascribed to *sp* ^3^ hybridized carbon and C–O–C/O–C = O bonding,^39,40^ probably formed due to the presence of saccharin in the electrolyte used for Ni electrodeposition. Adventitious carbon could contribute to the C 1s XPS spectrum as well; however, it is unlikely to account for 41.6% carbon in the coating (Table 1). The N 1s spectrum exhibited a single peak at 400.6 eV, which is likely due to surface contamination (the N content was below 4%).

**Figure 2:**
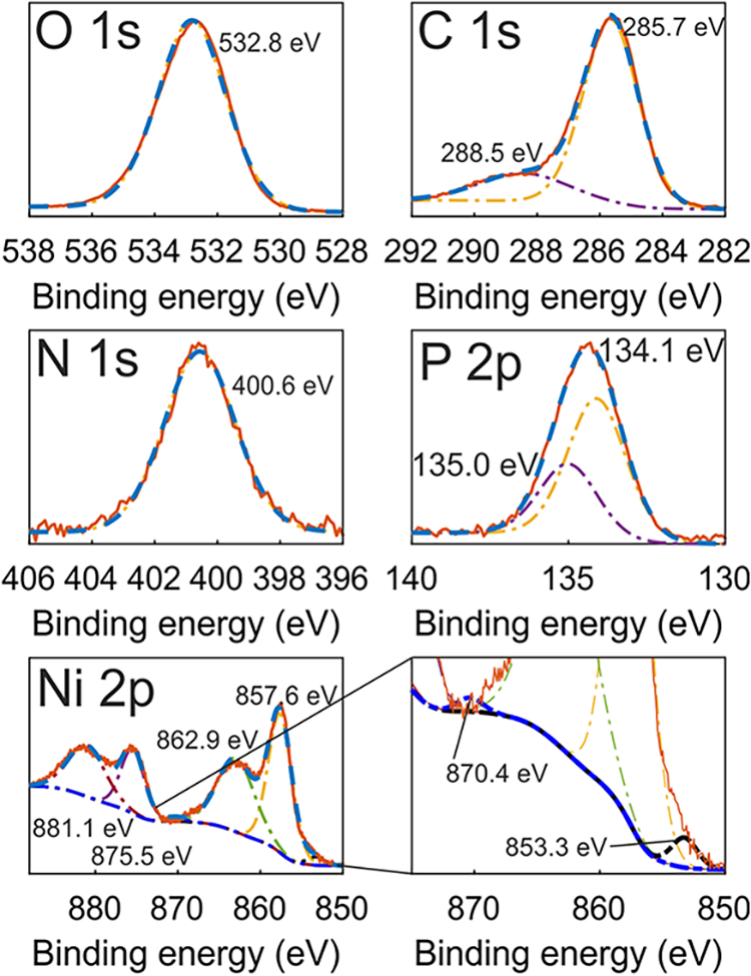
High-resolution XPS spectra for the b-Ni coating. Solid red lines represent the measured spectra, and dashed blue lines show the fitted envelopes. Dash-dotted lines represent the individual fitted peaks.

The P 2p spectrum contained two contributions peaked at 134.1 and 135.0 eV, associated with oxidized phosphorus (nickel phosphate and/or nickel hydrophosphate/dihydrophosphate).^41–43^ At the same time, Barbaux *et al*. have demonstrated slight variations in P 2p peak positions for various forms of phosphates (meta-, ortho-, di-, and polyphosphate).^44^ Thus, the resulting P 2p spectrum is associated with a mixed composition of phosphate products formed during the oxidation of the b-Ni coating with nitric acid.

The high-resolution Ni 2p spectrum contained three doublet components with 2p_3/2_ peaks at 853.3, 857.6, 862.9, and 2p_1/2_ peaks at 870.4, 875.5, and 881.1 eV. The doublet with peaks at 853.3 and 870.4 eV can be ascribed to metallic nickel.^45^ Compared to the standard binding energy of pure metallic Ni (852.8–853.1 eV), the position of the main Ni 2p_3/2_ peak (853.3 eV) of the b-Ni coatings shifted to higher binding energies, reflecting a strong interaction between Ni and P or C.^46^

The doublet at 857.6 and 875.5 eV can be assigned to overlapping Ni^2+^ and Ni^3+^ species in the Ni 2p_3/2_ and Ni 2p_1/2_ orbitals, respectively. The peak at 857.6 eV is substantially shifted upward compared to the values characteristic of NiO (854.8 eV), which excludes the presence of NiO in the coating. According to data from the literature,^47^ the coating can contain nickel hydroxide Ni­(OH)_2_, nickel metahydroxide NiOOH, nickel­(III) oxide Ni_2_O_3_, and nickel phosphate Ni_3_(PO_4_)_2_.

The doublet with 2p_3/2_ at 862.9 eV is possibly due to the overlap of the shakeup satellites of the peaks at 853.3 and 857.6 eV as well as peaks from Ni^3+^.^48^ Similarly, the peak at 881.1 eV is the 2p_1/2_ component of this satellite doublet.

### Light Absorption Properties of the Black Ni Coating

Following structural characterization, the light absorption performance of the b-Ni coating was evaluated across an ultrawide spectral range. Emission measurements were conducted in the 3–35 THz range and compared to a reference sample (inset, Figure 3). The reference consisted of a silicon moth-eye structure coated with pyrolytic carbon, previously reported as a near-perfect absorber in our earlier studies.^18,19^ In the frequency range below 25 THz, the b-Ni coating exhibited lower emission intensity than the reference. However, at frequencies above 25 THz, both samples demonstrated nearly identical emissive behavior.

**Figure 3:**
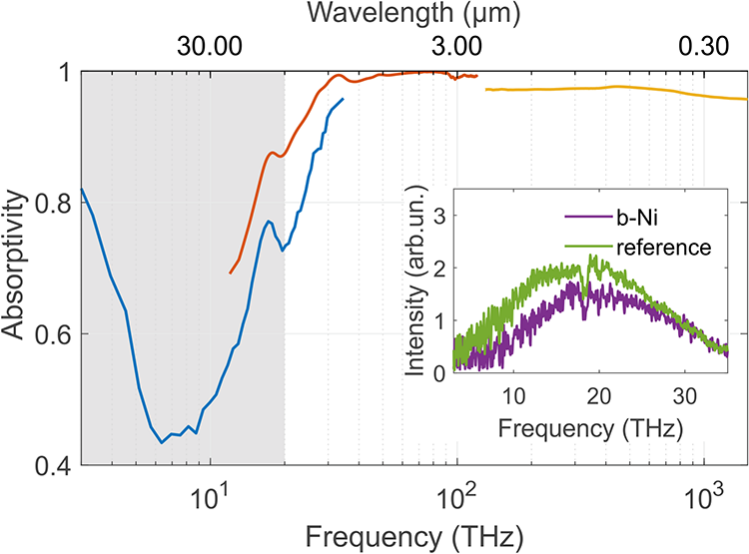
Absorptivity spectrum for the b-Ni coating. The spectrum in the 3–35 THz range (blue curve) was recalculated from the emission spectrum. The spectra in the 12–120 THz (red curve) and 130–1500 THz (yellow curve) were recalculated from the reflectivity spectra measured using FTIR and DRS techniques, respectively. The gray box highlights the dip in absorbance between 3 and 20 THz. The inset compares the emission spectra for the b-Ni coating (purple curve) and for the reference sample (Si moth-eye structure described in our previous work,^19^ green curve).

Emission values were subsequently converted to absorptivity using Kirchhoff’s law (see eq 1). At 3 THz, the b-Ni coating demonstrated strong absorption, exceeding 80% of incident radiation (Figure 3). Between 3 and 20 THz, a noticeable dip in absorptivity was observed, consistent with the lower emissivity of the b-Ni coating compared to the reference sample.

The reflectance spectrum of the b-Ni coating was measured across the 12–1500 THz range. The reflectivity values were converted to absorptivity using eq 2, assuming zero transmittance due to the presence of a 40 μm-thick copper backreflector. In the 30–120 THz range (FTIR measurements), the coating exhibited high absorptivity values between 0.985 and 0.999 (Figure 3). In the 130–1500 THz range (DRS measurements), absorptivity ranged from 0.958 to 0.972.

The slight discrepancy between the absorption measurements in different spectral ranges can be attributed to differences in the measurement geometry. More specifically, in the emissivity measurements (blue curve), the radiation was collected from directions diverging from the normal to the sample by up to 12°. Although diffusely reflected light was collected in both FTIR and DRS measurements, the detector geometries differed between the two techniques.

The high absorptivity of the b-Ni coating across an ultrabroadband frequency range can be attributed to its hierarchical structural organization. Micron-scale cone-shaped features and pores are likely responsible for efficient light absorption in the low-frequency regime (below ∼50 THz). At higher frequencies, the nanoscale roughness of the cone surfaces contributes significantly to absorption.

### Modeling of the Optical Response

Modeling was used to emulate the observed light absorption properties of the b-Ni coating. The simulations were performed using full-wave simulations in COMSOL Multiphysics with the geometry of the model discretized using a tetrahedron mesh (496,913 tetrahedrons), providing a balance between numerical accuracy and computational efficiency. The frequency domain simulations were performed using an interpolative frequency sweep with an error threshold of S parameter of 0.01, with a total of 1001 frequency data samples obtained.

First, the light absorption behavior was simulated in the low frequency range (up to 100 THz). The optical response in this range is likely dominated by the micron-sized cone-like structures of the b-Ni coating (Figure 1A–C). Hence, the unit cell (Figure 4A) consisted of a truncated Ni cone with electrical conductivity σ = 1.44 × 10^7^ S/m, with the bottom diameter set to 5 μm and the top diameter to 2.5 μm, while its height was set to 10 μm. A 4 μm Ni layer was used as the substrate. The unit-cell was simulated with periodic boundary conditions along the *x*- and *y*-axes, and the material was excited along the *z*-axis.

**Figure 4:**
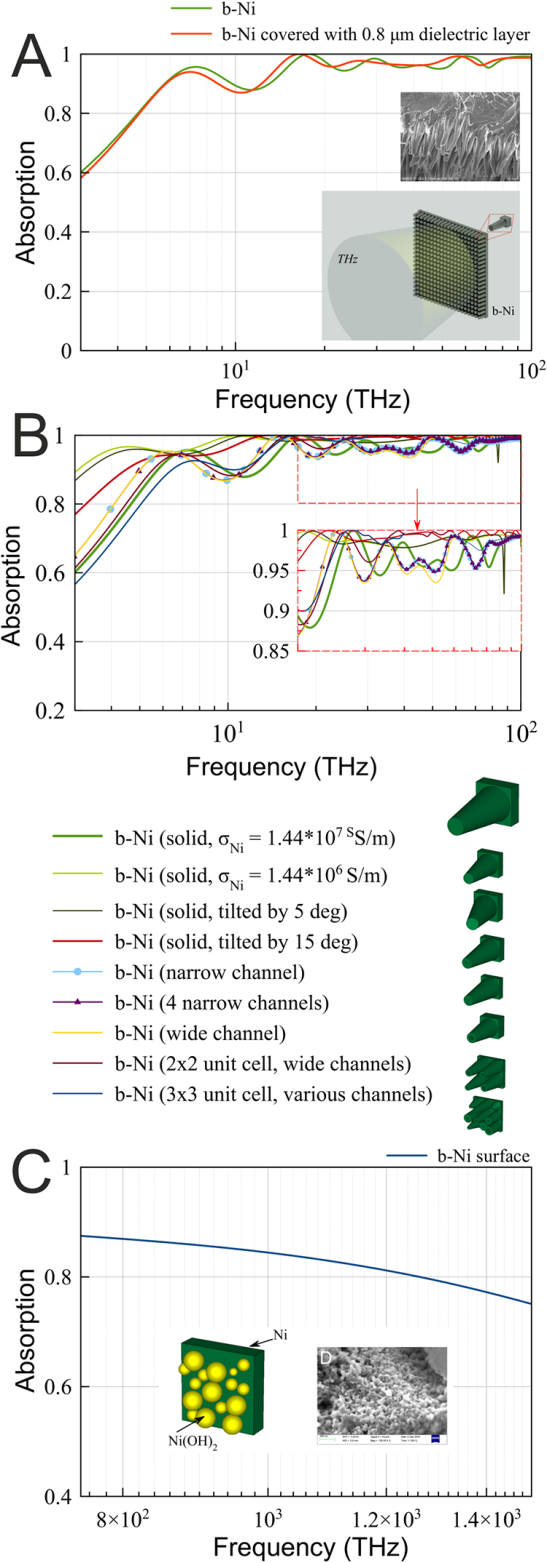
Simulated absorptivity spectra. (A) Spectrum in the low frequency range for the truncated cones made of Ni (green line) and for the same structure covered with a thin dielectric layer (red line). (B) Spectra in the low frequency range for various morphologies of the b-Ni. (C) Spectrum in the high frequency range for a surface layer comprising Ni­(OH)_2_ nanoparticles.

Given that the b-Ni coating is not purely metallic but is covered by an oxidized dielectric layer, we compare the simulated absorption spectrum for a periodic array of truncated Ni cones with and without a thin (0.8 μm) dielectric coating (Figure 4A).

Although this simplified structure allows one to reproduce the overall light absorption properties of the coating in the THz range with sufficient accuracy, both the dimensions of the structural features and the dielectric coating affect the absorption spectrum. In particular, the dielectric coating influences the position of local minor peaks (Figure 4A, red line). The morphology of the structure also plays a role: since the fabrication process is inherently random, there is some variation in the feature size, orientation, and slope. Therefore, each individual element has a slight influence on the THz response; however, when averaged over the entire structure, these variations do not significantly affect the overall absorption spectrum. To demonstrate this, eight variations of the truncated cone geometry of increasing complexity were simulated, all yielding comparable results (Figure 4B): all geometries exhibit broadband absorption across the investigated frequency range with similar spectral trends. Yet, each unit-cell geometry affects various features of the absorption spectrum, like the level of the absorption at lower frequencies or local minima positions.

These results indicate that the absorption behavior arises primarily from the microscale cone geometry and the periodicity of the array, while when averaged the contributions of the nanoscale roughness and cone-to-cone irregularities can be considered minimal. Therefore, the simplified solid truncated cone unit cell provides a reliable and computationally efficient representation of the fabricated structure.

The optical response in the higher frequency range (above 100 THz) is likely influenced by the nanoscale roughness of the b-Ni coating (Figure 1D). These structural features were simulated by a 1 μm Ni layer covered with randomly arranged within the unit cell Ni­(OH)_2_ particles as seen in Figure 4C. Although the absorptivity values of >80% achieved in the simulation are considerably lower than the experimental ones, the simulated geometry does not account for the larger cones and the nanoscale structures simultaneously.

Thus, the simulation results suggest that the absorption of the b-Ni coating in the low-frequency range is primarily due to the quasi-periodic array of micron-sized, cone-like structures. In contrast, the nanostructured surface of these cones likely accounts for absorption in the high-frequency range. While the simulations capture the general trends in light absorption, the use of simplified geometries may explain the discrepancies between the measured and simulated spectra. Additionally, multiple internal reflections within the cone-like structures could further enhance absorption, as previously demonstrated for moth-eye Si structures.^18^ These processes are schematically depicted in Figure 5.

**Figure 5:**
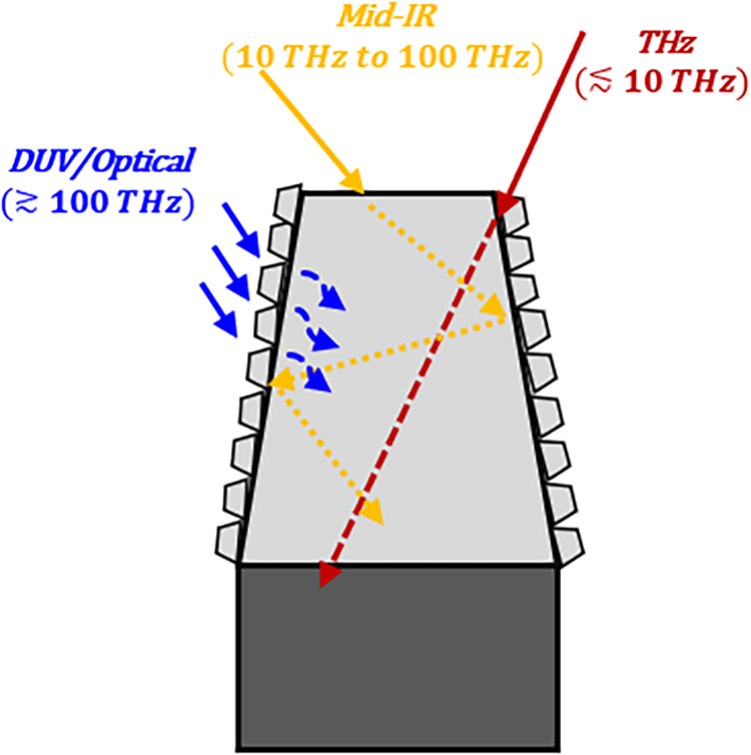
Schematic of frequency-dependent absorption in black Ni. Lower frequencies are absorbed via impedance matching and Ohmic losses, intermediate frequencies are trapped by multiple scattering in the microstructured porous cones, and higher frequencies are absorbed near the surface due the nanoscale roughness.

## Conclusions

The developed b-Ni coating demonstrates exceptional broadband absorption spanning an ultrawide frequency range from 30 to 1500 THz, achieved through a straightforward, scalable, and cost-effective fabrication process. This coating offers a robust, practical alternative to complex nanostructured absorbers like moth-eye or metamaterial designs, without compromising performance. Using a simple simulation approach, we demonstrate that the b-Ni coating can absorb light in an ultrawide spectral range due to its hierarchical structure consisting of micron-sized cones combined with nanoscale surface roughness. Its high absorptivity, mechanical durability, and chemical stability make it ideal for large-area applications, while the simple, versatile electrodeposition and etching process enables easy integration into existing scientific, industrial, and technological platforms. These features make the b-Ni coating a promising material for applications such as advanced scientific instruments, thermal management in quantum and photonic devices, electromagnetic shielding, and energy harvesting, supporting its use in next-generation broadband absorbers.

## Supplementary Material



## Data Availability

The data that support the findings (XPS spectra, measured and simulated absorptivity spectra) of this study are openly available in Zenodo at 10.5281/zenodo.17303895, reference number 17303895.

## Notes

EDS mapping of the black nickel sample (PDF)
