# Supplementary material for: Black Nickel Coating as a Broadband Terahertz to Deep-Ultraviolet Absorber
Source: ACS Omega. 2026 Feb 3;11(6):10723–9. doi: 10.1021/acsomega.5c12663 (PMC12917823; doi:10.1021/acsomega.5c12663)
Supplement: Supplementary file 1 [file ao5c12663_si_001.pdf]

Supporting Information

Black nickel coating as a broadband terahertz to deep-ultraviolet absorber

Aadya Menon[†*1], Hanna Maltanava[*2], Nikita Belko[2], Maria Cojocari[2], Mikhail Gorbun[2], Aleksandr Saushin[2], Konstantin Tamarov[3], Jari T.T. Leskinen[3], Mikko Selenius[3], Sari Suvanto[4], Dmitry Semenov[1], Sergei Malykhin[2], Vesa-Pekka Lehto[3], Georgy Fedorov[2], and Polina Kuzhir[2]

1. School of Computing, University of Eastern Finland, Joensuu, Finland
2. Department of Physics and Mathematics, University of Eastern Finland, Joensuu, Finland
3. Department of Technical Physics, University of Eastern Finland, Kuopio, Finland
4. Department of Chemistry and Sustainable Technology, University of Eastern Finland, Joensuu, Finland

† Email: aadya.menon@uef.fi

EDS mapping of the b-Ni coating

It can be seen that oxygen and phosphorus become more abundant when moving from the base to the tip of the cone-shaped structures.

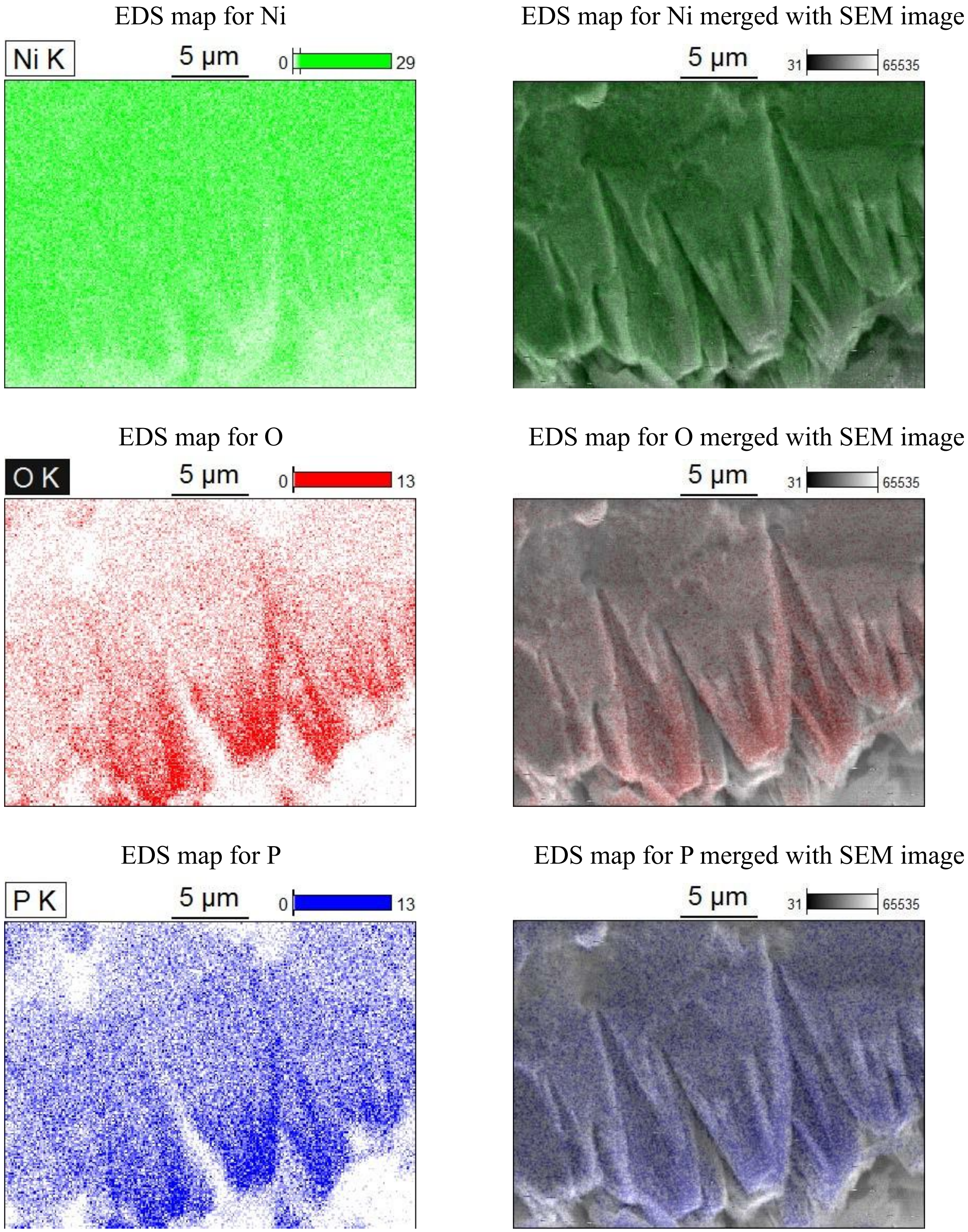

Fig. S1. EDS mapping of the elemental distribution in the b-Ni coating.
